# Switching anti-VEGF agent for wet AMD: evaluation of impact on visual acuity, treatment frequency and retinal morphology in a real-world clinical setting

**DOI:** 10.1007/s00417-020-05059-y

**Published:** 2021-01-07

**Authors:** Elisabet Granstam, Sandra Aurell, Kersti Sjövall, Anna Paul

**Affiliations:** 1 Centre for Clinical Research, Uppsala University/Region Västmanland, Västerås, Sweden; 2 Department of Ophthalmology, Region Västmanland, S-721 89 Västerås, Sweden

**Keywords:** Wet AMD, Anti-VEGF agent, Treatment, Switch, Visual acuity

## Abstract

**Purpose:**

The aim of the present cross-sectional real-world study is to evaluate the impact of switch of anti-VEGF agent from ranibizumab to aflibercept on visual acuity, treatment frequency and retinal morphology after 12 months in eyes with ongoing chronic treatment for wet age-related macular degeneration (AMD) compared to eyes not subjected to switch of anti-VEGF agent.

**Methods:**

Data was obtained retrospectively from the Swedish Macular Register, spectral-domain optical coherence tomography (OCT) images and electronic patient charts. All eyes included were treated in the same clinical setting at the Department of Ophthalmology at the county hospital of Västmanland in Västerås, Sweden.

**Results:**

In total, 282 and 359 eyes were included in the non-switch and switch cohorts, respectively. The cohorts were well balanced. Visual acuity remained stable during the observation period in both cohorts of eyes. The number of anti-VEGF treatments slowly declined over time in both cohorts of eyes and, consequently, the treatment intervals increased during the observation period. In eyes subjected to switch of anti-VEGF agent, planned treatment interval at 12 months was 7.6 (mean; SD 2.9) weeks compared to 6.8 (mean; SD 2.7) in the non-switch cohort (*P* = 0.001). OCT images demonstrated lower prevalence of intraretinal and subretinal fluid as well as pigment epithelial detachment at 12 months in eyes subjected to switch of anti-VEGF agent compared to non-switch eyes.

**Conclusion:**

Switch of anti-VEGF agent from ranibizumab to aflibercept did not affect visual function whereas improvement in retinal morphology was observed. These findings suggest a beneficial effect of switching from ranibizumab to aflibercept in eyes with ongoing chronic anti-VEGF treatment irrespective of previous response to ranibizumab. Longer follow-up is required to further evaluate the potential clinical significance of this finding.

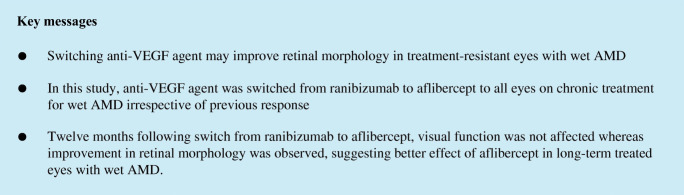

## Introduction

Age-related macular degeneration (AMD) is the leading cause of severe visual impairment in the elderly of the western world [1]. Wet AMD, which is caused by proliferating blood vessels under and within the retina in the macular region, accounts for 10–15% of all AMD [2]. Since 2006, the mainstay treatment for wet AMD has been repeated intravitreal administration of anti-vascular endothelial growth factor (anti-VEGF) agents [3, 4]. At present, three different anti-VEGF agents are approved for treatment of wet AMD: ranibizumab, aflibercept and brolucizumab [3–6]. In addition, bevacizumab is used off-label [7].

Different treatment algorithms have been applied for the administration of the anti-VEGF agents. Initially, monthly treatment was demonstrated to improve and stabilise visual acuity [3, 4]. Further on, comparable visual results were obtained using monthly clinical controls and treatment only in case of signs of disease activity in terms of reduction of visual acuity, increase in retinal macular oedema on optical coherence tomography (OCT) or new/persistent haemorrhage were identified (treatment as needed, pro re nata, PRN) [7]. Proactive treatment with gradually increased intervals between treatments (treat-and-extend, TE) was first described in 2015 (Berg et al.) [8]. We have previously reported better visual outcome with TE regimen compared to PRN regimen in a clinical real-world setting using ranibizumab [9].

Not all eyes respond favourably to anti-VEGF treatment [10]. Differences in pharmacological properties between anti-VEGF agents may account for interindividual differences in treatment response and switching between anti-VEGF agents has frequently been tried in treatment-resistant eyes [11, 12]. Development of tachyphylaxis towards the anti-VEGF agent has also been suggested as a potential mechanism for reduced treatment effect after chronic treatment [13]. Several studies have demonstrated that switching from one anti-VEGF agent to another in treatment-resistant eyes may lead to improvement in the anatomical outcome whereas changes in visual acuity are less consistent [12].

In our clinical setting, a switch of anti-VEGF agent from ranibizumab to aflibercept was instituted from an economical point of view. All eyes with ongoing treatment for wet AMD on November 1, 2017, were switched to aflibercept irrespective of previous response to ranibizumab. The aim of the present cross-sectional study was to evaluate the impact of the switch of anti-VEGF agent on visual acuity, treatment frequency and retinal morphology on OCT in eyes with ongoing chronic treatment for wet AMD.

## Materials and methods

This study is a retrospective, cross-sectional, observational study. Data was obtained from the Swedish Macular Register (SMR), spectral-domain OCT images from the database ImageNet (Topcon Corporation, Tokyo, Japan) and from electronic patient charts. The study was approved by the regional ethics committee of Uppsala/Örebro (Dnr 2018/521, approval date December 12, 2018) and adhered to the tenets of the 1964 Declaration of Helsinki and its later amendments. Each patient had approved the registration of medical data in the SMR and additional consent for participation in the present study was not required as approved by the regional ethics committee. All eyes included in the study were treated for wet AMD in the same clinical setting at the Department of Ophthalmology at the county hospital of Västmanland in Västerås, Sweden.

All treatment for wet AMD given at the department is registered in the SMR. The registry was used to identify two cohorts: one cohort of eyes with a registered visit between October 1, 2016, and January 31, 2017 (non-switch cohort) and one cohort of eyes with a registered visit between October 1, 2017, and January 31, 2018 (switch cohort). This visit was defined as the index visit. For eyes to be included in the study, it was required to have received anti-VEGF treatment for wet AMD according to the TE regimen applied at the department for a minimum of 1 year before the index visit and had been followed up for a minimum of 1 year after the index visit. Additional exclusion criteria consisted of other treatment regimens than TE at the time of the index visit, switch from ranibizumab to aflibercept before the index visit or after the index visit but before 12 months of follow-up. Data regarding patient demography (age, visual acuity, type of macular neovascularisation and duration of anti-VEGF treatment at index visit) was collected from the SMR whereas data regarding treatment (treatment regimen, number of anti-VEGF injections, anti-VEGF agent and treatment interval at 12 months) was collected from electronic patient charts.

In the TE regimen applied at the department, patients are treated every 4 weeks until no signs of active wet AMD as determined by OCT are found. In eyes without signs of active disease, an anti-VEGF agent is administered and the interval to the next treatment is then extended by 2 weeks at a time up to a maximum interval of 12 weeks. If signs of recurrent disease occur, defined as reduction in visual acuity ≥ 5 letters on the Early Treatment Diabetic Retinopathy Study (ETDRS) letter chart, occurrence of intraretinal fluid (IRF) or subretinal fluid (SRF) on OCT and/or new or persistent haemorrhage, treatment interval is shortened by 2 weeks at a time until the disease again is considered to be inactive. Eyes remain on that interval for at least 6 months before treatment interval is extended again, as described above. After anti-VEGF treatment at 12-week treatment interval has been provided four times, active treatment is withheld and assessment of visual acuity and OCT is continued. Treatment is resumed in case of disease activity, pro re nata (PRN).

OCT images were collected from the OCT database ImageNet. The fast macular thickness map protocol was applied using the OCT 2000 instrument (Topcon Corporation, Tokyo, Japan). Retinal morphology was evaluated from OCT images taken at the index visit or at the visit immediately before this time point and from OCT images taken 12 months after the index visit or at the visit immediately before this time point. The presence or absence of SRF, IRF and pigment epithelial detachment (PED) was evaluated by two of the authors (EG, SA). The variables were defined according to Schmidt-Erfurth et al. [14]. Additionally, central retinal thickness (CRT) was measured at the same time points.

The primary outcome was visual acuity at the end of the observation period (12 months after switch of drugs from ranibizumab to aflibercept or after 12 months of continued treatment with ranibizumab). Visual acuity was measured with the ETDRS letter chart at 2 m. In a few cases, visual acuity at treatment start was measured using the Snellen chart and was converted to ETDRS according to Gregori et al. [15]. Secondary outcomes were number of anti-VEGF injections, treatment interval at 12 months and proportion of eyes with IRF, SRF and PED at 12 months. Statistical analyses were performed using SPSS 26 (IBM Corporation, Armonk, NY, USA). Student’s *t* test for paired and unpaired data was applied as appropriate. Association between OCT findings at the index visit and visual acuity at 12 months and association between VA and CRT at treatment start, duration of anti-VEGF treatment at an index time point, presence of IRF, SRF and PED and visual acuity at 12 months was investigated with linear regression analysis. *P* value < 0.05 was considered statistically significant.

## Results

**Fig. 1:**
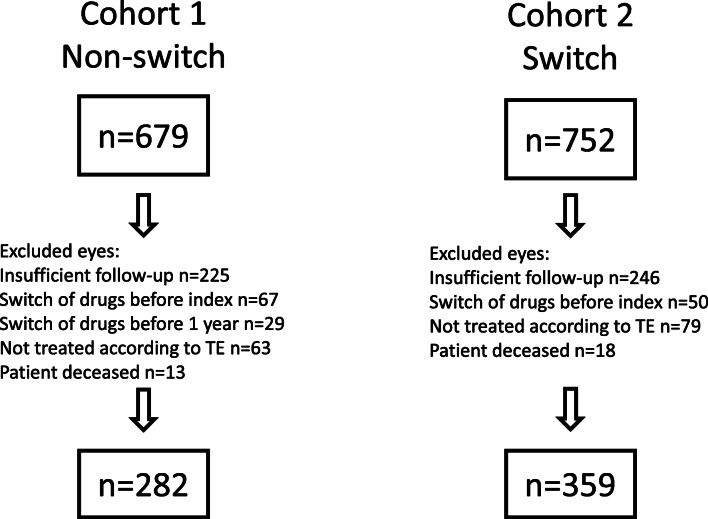
Inclusion of study eyes. TE, treat-and-extend treatment regimen

In total, 679 eyes (581 individuals) in the non-switch cohort and 752 eyes (635 individuals) in the switch cohort were identified in the SMR, respectively. Almost one-third of eyes in each cohort were excluded due to insufficient duration of treatment before the index visit or follow-up less than 12 months after index visit. After additional exclusion criteria had been adopted, the remaining 282 eyes in non-switch cohort and 359 eyes in switch cohort were included in the study (Fig. 1).

### Demography

Demographic characteristics of the two cohorts are presented in Table 1. Characteristics were well balanced between cohorts with regard to age and visual acuity. Among included patients, 65% were females in both groups. Eyes in both cohorts had received anti-VEGF treatment for wet AMD during approximately 4 years (non-switch cohort 43 months, and switch cohort 47 months) before index visit (Table 1). The proportion of eyes which had been in treatment 24 months or less was somewhat larger in the non-switch cohort compared to the switch group. IRF was slightly more prevalent in the non-switch cohort compared to the switch cohort at the start of anti-VEGF treatment (Table 1).

**Table 1:** Demographic data for the study cohorts. Visual acuity (ETDRS letter score), anatomical data regarding proportion of eyes (%) with intraretinal fluid (IRF), subretinal fluid (SRF), pigment epithelial detachment (PED), central retinal thickness (CRT, μm) at the start of anti-VEGF treatment (baseline, BL) and proportion of eyes (%) with type 1 (occult) and type 2 (classic) macular neovascularisation in the two cohorts at BL. Age (years), visual acuity (ETDRS letter score) and duration of anti-VEGF treatment for wet AMD at the index visit time point are presented. Continuous variables are given as mean and standard deviation. Student’s *t* test for unpaired data was applied

	Non-switch cohort, *n* = 282	Switch cohort, *n* = 359	*P* value
Visual acuity at BL (ETDRS letter score)	62.4 (12.7)	63.8 (12.6)	ns
IRF (%)	71	60	*P* = 0.005
SRF (%)	88	91	ns
PED (%)	70	70	ns
CRT (μm)	308 (104)	310 (97)	ns
Type 1 macular neovascularisation (%)	41 (74/179)	41 (78/190)	ns
Type 2 macular neovascularisation (%)	53 (94/179)	53 (100/190)	ns
Age at index visit (years)	80.7 (7.6)	80.6 (7.8)	ns
Gender (female, %)	65.6	65.2	ns
Visual acuity at index visit (ETDRS letter score)	65.8 (13.2)	66.9 (13.1)	ns
Duration of anti-VEGF treatment at index visit (months)	43 (27)	47 (28)	ns
Anti-VEGF treatment 24 months or less at index visit (%)	35	28	*P* = 0.000

### Visual acuity

**Fig. 2:**
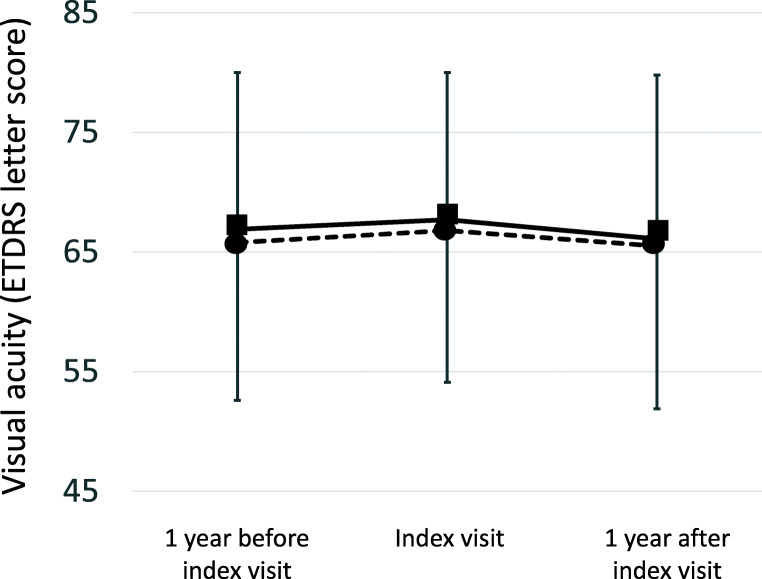
Visual acuity on ETDRS (Early Treatment Diabetic Retinopathy Study) letter chart (mean, standard deviation) at the index visit, at 12 months before and at 12 months after the index visit in the two cohorts. Broken line, non-switch cohort; continuous line, switch cohort

Visual acuity (ETDRS letter score) increased significantly from treatment start in both cohorts (*P* = 0.000) and then remained stable during the observation period (Fig. 2, Table 2). The mean improvement in visual acuity from treatment start to index time point was 4.3 (SD 12.3) and 4.1 (SD 13.5) letters ETDRS in the non-switch and switch cohort, respectively.

**Table 2:** Visual acuity (ETDRS letter score) at treatment start (baseline), at 12 months before index, at index time point and 12 months after the index time point in the two cohorts (mean, standard deviation). Student’s *t* test for unpaired data was applied

	Non-switch cohort, *n* = 282	Switch cohort, *n* = 359	*P* value
Treatment start	62.4 (12.7)	63.8 (12.6)	ns
12 months before index time point	65.9 (12.7)	67.5 (11.7)	ns
Index time point	66.6 (12.9)	67.7 (12.3)	ns
12 months after index time point	65.4 (13.7)	66.1 (13.7)	ns

### Anti-VEGF treatment

**Fig. 3:**
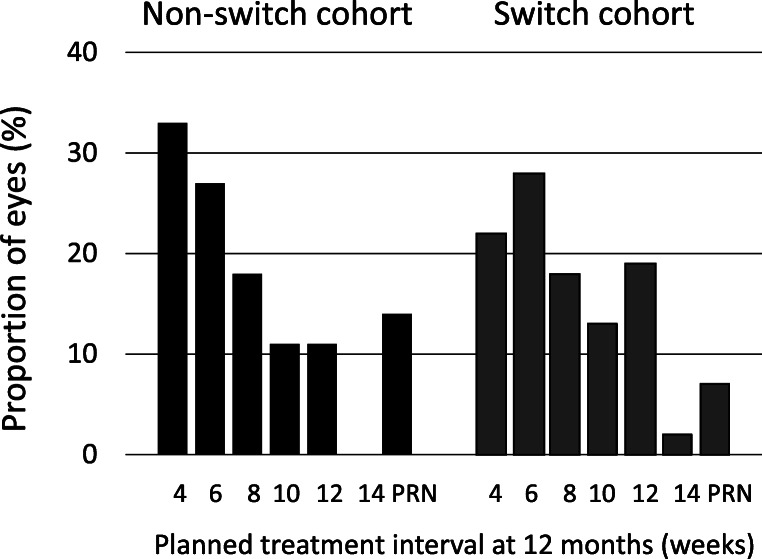
Planned treatment interval (weeks) at 12 months in the non-switch and switch cohorts, respectively. PRN, pro re nata*.* Proportion of eyes (%)

In the non-switch cohort, a mean of 8.5 (SD 2.7) ranibizumab injections were administered during the year before the index visit and 7.1 (SD 3.2) ranibizumab injections during the year following the index visit. The corresponding figures for the switch cohort were a mean of 9.0 (SD 2.8) ranibizumab injections before the index visit and 7.0 (SD 2.7) aflibercept injections during the year following the index visit. The number of intravitreal injections during the year following the index visit was not statistically significantly different between cohorts (*P* = 0.873). The number of anti-VEGF injections was significantly reduced over time in both cohorts (*P* = 0.000). Planned treatment intervals were 6.4 (SD 2.4) weeks, 7.3 (SD 3.1) weeks and 6.8 (SD 2.7) weeks in the non-switch cohort at 12 months before the index visit, at the index visit and 12 months after the index visit, respectively. The corresponding figures for the switch cohort were 6.0 (SD 2.4) weeks, 6.9 (SD 2.7) weeks and 7.6 (SD 2.9) weeks. The planned treatment interval at 12 months after the index visit was significantly longer in the switch cohort compared to the non-switch cohort (*P* = 0.001). Treatment intervals at 12 months are shown in Fig. 3. A minor proportion of eyes in each cohort continued on a *PRN* regimen in accordance with the treatment strategy applied at the department.

### OCT

**Fig. 4:**
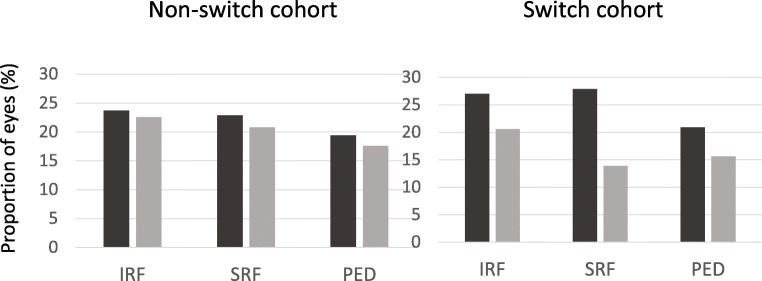
Presence of intraretinal fluid (IRF), subretinal fluid (SRF) and pigment epithelial detachment (PED) at the index time point (black bars) and at 12 months after index (grey bars) in the non-switch and switch cohorts, respectively. Proportion of eyes (%)

Qualitative evaluation of OCT images obtained at the index visit and at 12 months was performed. OCT data was not available for three eyes in the non-switch cohort. At the index time point, IRF and SRF were present in one-fourth of eyes in both cohorts, whereas PED was less frequent (Fig. 4). There was no difference in prevalence of IRF or PED between the two cohorts whereas SRF was more prevalent in the switch cohort compared to the non-switch group (28% compared to 23% of eyes; *P* = 004). In the non-switch cohort, the prevalence of IRF, SRF and PED remained unchanged 12 months after index visit (Fig. 4). However, in the switch cohort, fluid in all three compartments (IRF, SRF and PED) was less prevalent after 1 year of aflibercept treatment (Fig. 4). Mean CRT remained unchanged from the index visit to 12 months after index visit in the non-switch cohort: 208 (SD 54) compared to 207 (SD 61) μm (*P* = 0.643). The mean CRT was significantly reduced from index visit to 12 months after index visit in the switch cohort: from 212 (SD 54) to 201 (SD 51) μm (*P* = 0.000). Linear regression analysis demonstrated a positive association between SRF at the index visit and better visual acuity 12 months after index visit (*P* = 0.020). Neither presence of IRF or PED at the index visit was associated with visual acuity outcome 12 months after index visit.

### Regression analyses

Multiple linear regression analysis demonstrated a positive association between better visual acuity at treatment start as well as shorter duration of anti-VEGF treatment and better visual outcome at 12 months irrespective of switch (*P* = 0.000) whereas there was a negative association between presence of PED at treatment start and visual acuity at 12 months (*P* = 0.010). There was no association between IRF, SRF and CRT at treatment start and visual outcome at 12 months for the combined cohorts. Subgroup analysis in the non-switch and switch cohorts confirmed the positive association between visual acuity at treatment start and shorter duration of anti-VEGF treatment and better visual outcome at 12 months (*P* = 0.000).

## Discussion

In the present study in eyes with ongoing chronic anti-VEGF treatment for wet AMD, we found that visual function was stable during the observation period both in eyes subjected to switch of anti-VEGF agent from ranibizumab to aflibercept as well as in eyes on continued ranibizumab treatment. The number of anti-VEGF injections slowly declined over time in both cohorts of eyes and, consequently, the treatment intervals increased during the observation period. In eyes subjected to switch from ranibizumab to aflibercept, planned treatment interval at 12 months was longer and macula was dryer compared to eyes not subjected to switch, indicating an improvement in the retinal morphology after the switch from ranibizumab to aflibercept.

Switch of anti-VEGF agent has been tried mainly in eyes responding poorly to the first anti-VEGF drug given. In a review of 38 switch studies, it was found that following a switch of anti-VEGF agent, anatomical improvement can often be demonstrated in previously treatment-resistant eyes [12]. In the present study, the switch of anti-VEGF agent was imposed on all eyes on chronic anti-VEGF treatment, regardless of initial response to the first anti-VEGF agent given (ranibizumab). Nevertheless, even in this setting, we demonstrated an improvement in both qualitative and quantitative OCT findings 12 months after switch from ranibizumab to aflibercept.

The effect of a switch of anti-VEGF agent in treatment-resistant eyes with wet AMD on visual function has been found to be more variable [12]. Stable or declining visual acuity has been observed in several retrospective switch studies [12], which is in line with the findings of the present cross-sectional study of eyes with chronic anti-VEGF treatment for wet AMD. In contrast, in a prospective study in 49 patients, improvement in visual acuity 6 months after switch from ranibizumab to aflibercept was demonstrated [16]. On the other hand, in another small controlled prospective study with 12-month follow-up in eyes requiring 4-weekly anti-VEGF injections, visual acuity and OCT findings were similar in both switch and non-switch group [17].

Ranibizumab is a small recombinant humanised monoclonal antibody Fab fragment which can neutralise all known active isoforms of VEGF [3, 4]. Aflibercept is a somewhat larger recombinant fusion protein, which can bind not only to VEGF but also to placental growth factor [5]. Differences in molecular size and pharmacokinetic plus pharmacodynamic properties might account for the interindividual differences in response to the two anti-VEGF agents and have been used as an argument for switching agent in non-responsive eyes. It has also been suggested that improvement after switch of agent might be caused by increased treatment intensity after a switch. However, this was not the case in our study as we demonstrated that treatment frequency declined over time in both non-switch and switch cohorts.

In the present study, we found an association between presence of SRF at the index visit and better visual acuity 12 months later, which is well in line with findings from the CATT study [18]. Fluid in other compartments of the retina, especially IRF, has been found to be harmful for retinal function [7, 18]. Interestingly, in a retrospective review following switch to aflibercept of 45 non-responsive eyes initially treated with ranibizumab, baseline SRF predicted shorter recurrence-free treatment interval whereas baseline IRF predicted longer recurrence-free treatment interval at 12 months [19] indicating a complex relationship between fluid, disease activity and treatment intensity in eyes with ongoing anti-VEGF treatment for wet AMD.

In the present study, the number of anti-VEGF injections given declined and the planned treatment intervals increased over time in both the switch and the non-switch cohort, suggesting a reduced need for anti-VEGF treatment during the observation period for all eyes regardless of anti-VEGF agent. Similar findings have been reported from long-term follow-up of the original pivotal ranibizumab studies [20] as well as from the CATT study [21]. Five years after start of the CATT study, 83% of eyes showed macular fluid on OCT [21]. As long as 7 years after treatment initiation in the pivotal ANCHOR and MARINA studies, 68% of eyes had signs of active exudative disease and 46% of eyes were on ongoing anti-VEGF treatment [20] emphasising the chronicity of wet AMD.

Regression analysis of all included eyes demonstrated that better visual acuity at treatment start was associated with better visual outcome over time irrespective of switch. This is well in line with other studies including data from the SMR [22]. Results from our study also found and association between shorter duration of anti-VEGF treatment and better visual outcome over time regardless of switch of drugs, indicating that less chronic eyes had a better visual function. The larger proportion of eyes with less than 24 months of treatment duration in the non-switch cohort might have incurred a favourable visual result in this group, but there were no such findings in the analyses. It is well established that visual acuity declines over time in spite of continuous anti-VEGF treatment [22, 23] as a result of degenerative changes in the retina due to chronic macular disease.

One strength of the present study is the cross-sectional study design allowing for comparison between eyes in two cohorts, one non-switch cohort and one cohort of eyes subjected to switch of anti-VEGF agent. Another strength is the well-described clinical setting [9], few physicians involved in the care of the wet AMD patients and good adherence to local guidelines. One weakness of the study is the lack of prospective data and the historical control group. A prospective clinical trial with randomisation to either continued ranibizumab treatment or switch to aflibercept would have been preferred but was not achievable in our real-world setting.

In conclusion, in our study of eyes with ongoing chronic anti-VEGF treatment for wet AMD, we found that switch of anti-VEGF agent from ranibizumab to aflibercept did not affect visual function whereas improvement in retinal morphology was observed and planned treatment intervals were prolonged. The findings suggest a beneficial effect of switching from ranibizumab to aflibercept in eyes with ongoing chronic anti-VEGF treatment for wet AMD irrespective of previous response to ranibizumab. Longer follow-up is required to further evaluate the potential clinical significance of this finding.

## Data Availability

All data is available through the SMR and upon request from Region Västmanland, Västerås, Sweden.
